# Plasmodesmata wall biomechanics: challenges and opportunities

**DOI:** 10.1093/jxb/eraf392

**Published:** 2025-09-04

**Authors:** Brigita Simonaviciene, Emily Newcombe, Alex Gresty, Yoselin Benitez-Alfonso

**Affiliations:** Centre for Plant Sciences, Bragg Centre for Materials Research and the Astbury Centre, School of Biology, University of Leeds, Leeds LS2 9JT, UK; Centre for Plant Sciences, Bragg Centre for Materials Research and the Astbury Centre, School of Biology, University of Leeds, Leeds LS2 9JT, UK; School of Physics and Astronomy, Faculty of Engineering and Physical Sciences, University of Leeds, Leeds LS2 9JT, UK; Centre for Plant Sciences, Bragg Centre for Materials Research and the Astbury Centre, School of Biology, University of Leeds, Leeds LS2 9JT, UK; University of Wisconsin, Madison, USA

**Keywords:** Cell wall biophysics, cell wall proteins, cellulose, intercellular connectivity, pectin, plant biomechanics, plasmodesmata transport, symplasmic pathway, wall permeability, xyloglucan

## Abstract

Plant cell walls exist as a complex and varied blend of polysaccharides and proteins; the combination of which has evolved over millions of years. Research on how these components interact is key to understanding a plant’s mechanical, structural, communicative, and biological traits. However, knowledge on cell wall components, its biophysical properties and cellular functions, remains sparse. Particularly challenging is the analysis of cell wall microdomains such as plasmodesmata. Plasmodesmata are membranous bridges embedded in cell walls facilitating cytoplasm-to-cytoplasm (i.e. symplasmic) transport of diverse factors, including proteins and signalling molecules that control plant development. Here, we review recent research on plasmodesmata cell walls connecting structural and mechanical properties of their components and evidence of their function at plasmodesmata. Most work in this area focuses on callose (a β-1,3-glucan that accumulates at plasmodesmata), but compositional and proteomic analysis indicate interplay with wall pectins, xyloglucans, and cellulose structures that remains under-investigated. We discuss the importance of understanding polymer interactions at the molecular and biophysical level, and their relevance for plasmodesmata biomechanics. We also highlight new techniques and outstanding questions and reflect on the opportunities for translation of knowledge in the improvement of plant traits and in biomaterial design.

## Teaser

The aim of this review is to highlight current research on plasmodesmata cell walls and their biomechanical properties and discuss the challenges and opportunities of parametrizing and dissecting their function.

## Introduction

The structure and physico-mechanical properties of cell walls play a crucial role in plant biology; influencing key processes such as cell division, organ shape, water and nutrient transport, cell-to-cell adhesion, and signalling (for recent reviews see Coen and Cosgrove, 2023; Cosgrove, 2024). Due to the incredible complexity of cell wall biology, a comprehensive understanding of how evolutionary and developmental variations in cell wall composition affects their function remains elusive. This knowledge is vital in the design of approaches to improve plant development and responses to environmental cues. It will also help inspire innovations that utilize plant cell wall resources in the biopolymer industries, accelerating the switch to renewable and biodegradable materials.

**Fig. 1.**
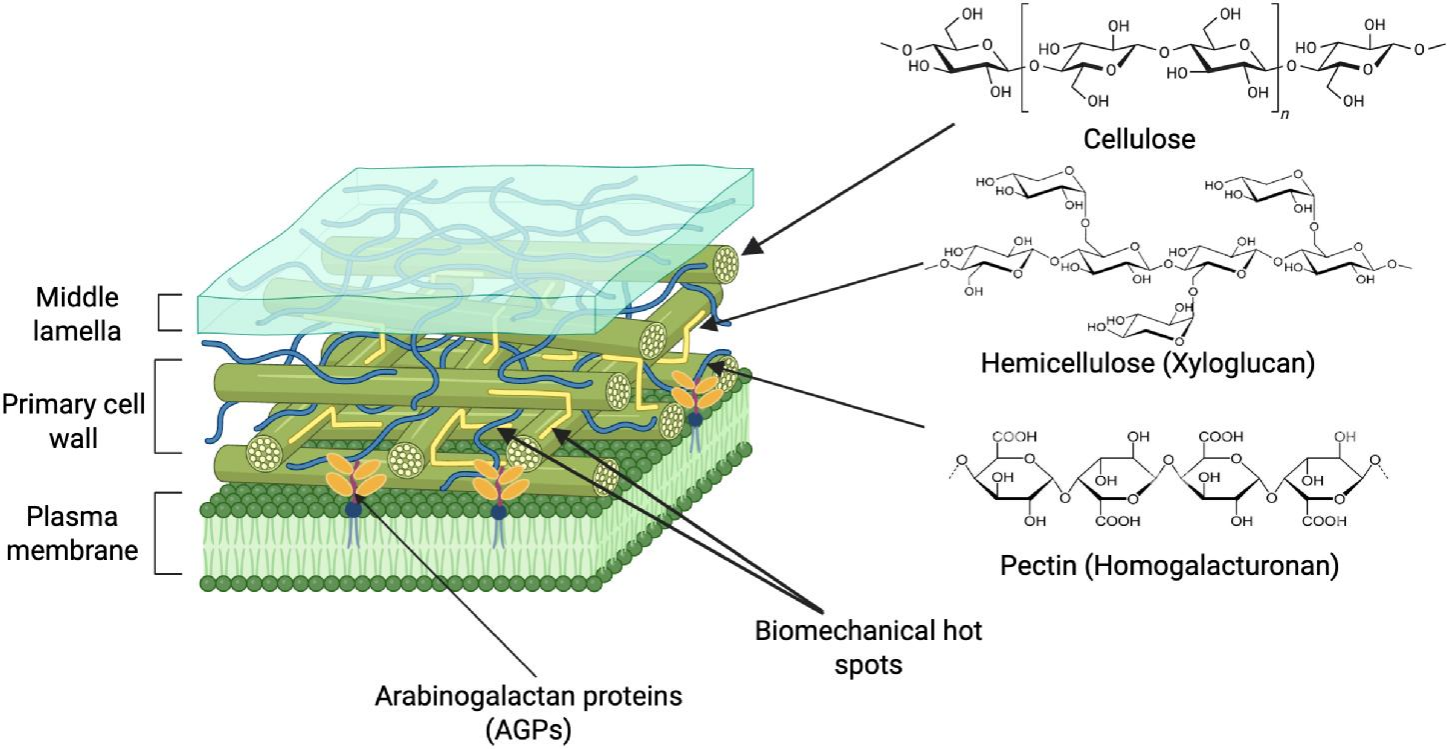
A simplified model of the primary cell wall structure in plants. The figure represents the extracellular space between developing cells, including the middle lamella, primary cell wall, and plasma membrane. The primary cell wall consists of cellulose microfibrils, hemicellulose (which includes xyloglucan, mannoglycans, xylan, mixed linkage β-glucans, and others), pectins (homogalacturonan, xylogalacturonan, rhamnogalacturonan I, rhamnogalacturonan II), and proteins (including arabinogalactan proteins, AGPs). The chemical structures of cellulose, xyloglucan, and the pectin homogalacturonan exemplify the diversity of these cell wall components. Biomechanical hotspots (labelled with arrows) are areas where pectins and xyloglucans interact and bind cellulose microfibrils. These hotspots control cell wall extensibility and biomechanics. (Created in Biorender. Simonaviciene, B. (2025) https://BioRender.com/jmcz0e7. The figure was adapted from Huang, E. (2025). Plant Cell Wall Structure. https://app.biorender.com/biorender-templates/details/t-65bafb2a93d8a4629e909333-plant-cell-wall-structure).

The composition of cell walls varies between plant species, and between organs, tissues, and even in microdomains within the same cell (Knox and Benitez-Alfonso, 2014; Cosgrove, 2016; Dauphin *et al.*, 2022; Gu and Rasmussen, 2022). Cell walls are classified as primary and secondary according to their biogenesis. Primary cell walls are secreted during cell division and contain a mixture of cellulose, hemicelluloses (including xyloglucans and mannans), pectins [named homogalacturonan (HG), rhamnogalacturonan (RG) I and II] and a few glycoproteins that interact to create biomechanical hotspots (Fig. 1, see Table 1 for definition) (Cosgrove, 2016; Gu and Rasmussen, 2022). Primary cell walls of neighbouring cells are joined by a pectic layer known as the middle lamella, which plays a significant role in maintaining cell adhesion (Zamil and Geitmann, 2017). Secondary cell walls are deposited after the cell ceases growing, and contain lignins which are complex 3D branched polymers of phenylpropane units that reinforce the cellulose microfibril network (Klinke *et al.*, 2001). The concentration, chemical structure, and interactions between wall components change dynamically during development (Gibson, 2012) and in response to environmental cues (Alonso Baez and Bacete, 2023). Structural features such as molecular substitutions and/or changes in biopolymer length (or degree of polymerization, Table 1) affect cell wall function as a biomechanical construct (Johnson *et al.*, 2018). Cell walls also act as biosensors changing their composition and eliciting mechanisms to adapt and respond to biotic and abiotic stresses. Cell wall integrity sensing mechanisms, involving receptor-like kinases such as FERONIA (FER), perceive these cues, modulate downstream signalling pathways, and adjust cell wall composition and mechanics (Tang *et al.*, 2022; Cheung, 2024).

**Table 1.** Definition of keywords associated with plant cell wall structural and mechanical properties

Terminology	Definition
Biomechanical hotspot	Localized load-bearing regions in cell walls where cellulose molecules are joined together via interactions with other cell wall components (mainly pectin and xyloglucan)
Cellulose microfibril	Cellulose chains linked together via hydrogen bonds into a fibre-like strand
Turgor pressure	Hydrostatic force that pushes the plasma membrane against the cell wall
Microfibril angle	Angle between cellulose microfibrils and the longitudinal axis of the cell wall
Degree of crystallinity	Fraction of cellulose that is crystalline/highly organised in structure
Degree of polymerization	Number of monomer units within a polymer chain
Stiffness	Mechanical strength referring to the resistance to deform when a force is applied
Elasticity	Capacity of cell walls to deform under mechanical stress and return to their original shape once the stress is removed
Softness	Reduction in cell wall stiffness mainly due to cell wall disassembly/loosening
Rigidity	Ability of the cell wall to withstand deformation from both internal and external forces and to maintain a fixed shape
Extensibility	Measure of cell wall ability to experience irreversible deformation increasing the surface area during growth
Plasticity	Irreversible deformation of the cell wall causing changes in shape and cell expansion
Ductility	Ability of the cell wall to withstand plastic deformation before fracturing

Plant biomechanics is a discipline at the interface between biology, physics, and mathematics dedicated to the study of the physical principles and processes that determine the structure and function of plant cells, tissues, and organs (Geitmann *et al.*, 2019). Cell wall biomechanical behaviour is dependent on their structural composition (biochemistry), on intracellular factors such as turgor pressure (defined in Table 1) and extracellular factors such as soil compaction, wind, or temperature, which influence cell wall architecture (Johnson *et al.*, 2018). Understanding the synergies between cell wall structural features (e.g. composition, microfibril angle, degree of crystallinity and polymerization, Table 1) and physico-mechanical properties (i.e. stiffness, elasticity, softness, rigidity, extensibility, plasticity, ductility, see Table 1) is the main goal of years of research. In this context, research on cellulose has found that microfibril structural organization (i.e. crystallinity), modulated by the presence of hemicelluloses and pectic components, regulates the stiffness and extensibility of cell walls (Cosgrove, 2022 and references therein). In addition, changes in cellulose microfibril orientation define the properties of cell wall microdomains (i.e. specific regions within cell walls with distinct composition, architecture, and function) (Dauphin *et al.*, 2022).

**Fig. 2.**
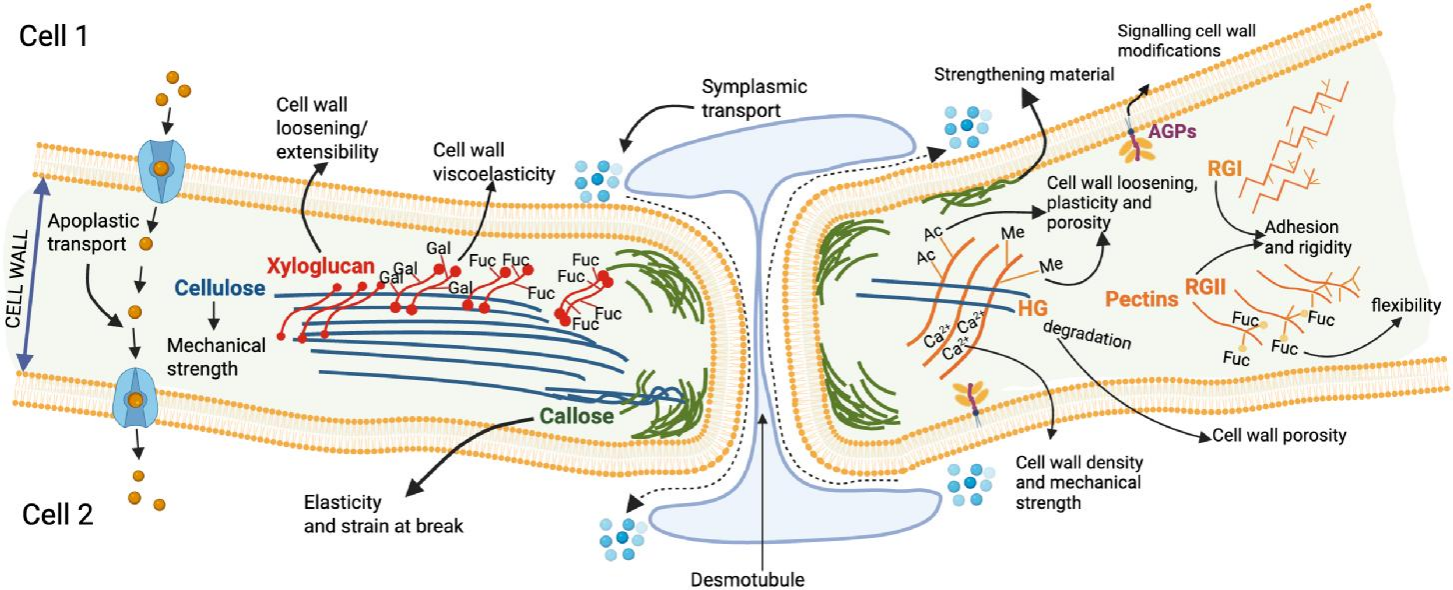
Model representing the mechanics of primary cell wall components and intercellular transport pathways. The figure shows plant molecular signals (as spheres) moving between neighbouring cells using the apoplastic (facilitated by import/export membrane carriers and cell wall diffusion of small molecules) or the symplasmic (mediated by plasmodesmata which are pores in the wall, traversed by a desmotubule, enabling cytoplasm-to-cytoplasm connections) transport pathways. The figure also displays cell wall components: cellulose microfibrils (blue rods), xyloglucans (in red), callose (in green around plasmodesmata), cell wall proteins (e.g. arabinogalactan proteins, AGPs) and various pectins [homogalacturonans (HGs) and rhamnogalacturonans (RGI and RGII)]. It also shows examples of structural modifications, such as addition of Fucosyl (Fuc), Galactosyl (Gal), Methyl (Me), or Acetyl (Ac) residues in xyloglucans and pectins, and the interactions of HG with calcium ions affecting their mechanical behaviour. Mechanical properties of each of these components are assigned based on publications reviewed in this paper. Table 2 elaborates on the properties of specific components linked to plasmodesmata regulation. (Created in Biorender. Simonaviciene, B. (2025) https://BioRender.com/v6wcgtf).

In plant cells, cell wall microdomains are associated with regions of connectivity, polar or apical cell expansion, and anisotropic growth (Fujita and Wasteneys, 2014; Dauphin *et al.*, 2022). Specialized cell wall microdomains characterized by cellulose microfibrils with distinct circular patterns surround plasmodesmata (singular plasmodesma, Fig. 2): membrane-lined channels, of approximately 50 nm in diameter, formed primarily during cytokinesis and connecting neighbouring cells (Z. P. Li *et al.*, 2021). Plasmodesmata generate a continuum of the plasma membrane (PM) forming a channel traversed by the desmotubule (a tubular structure made of modified endoplasmic reticulum, ER). Between the PM and the desmotubule, a cytoplasmic sleeve (named the symplasm) is defined, which facilitates the molecular transport of small and large molecules (including proteins, hormones, and RNAs) (Bayer and Benitez-Alfonso, 2024; Tee and Faulkner, 2024; Zanini and Burch-Smith, 2024). Overcoming the cell wall’s constraints, plasmodesmata can also form *de novo* (named secondary plasmodesmata) and/or suffer structural modifications post-cytokinesis (Nicolas *et al.*, 2017; Schreiber *et al.*, 2024; Wegner and Ehlers, 2024). Branched, funnel, and twinned structures, as well as changes in plasmodesmata density, are observed during tissue/organ development (Tee and Faulkner, 2024). Cell wall extension can also lead to the formation of pit-fields (clusters of interconnected plasmodesmata forming large pores) in specialized organs such as developing fruits (Orfila and Knox, 2000; Faulkner *et al.*, 2008).

**Table 2.** Cell wall components associated with changes in plasmodesmata (PD) function. The table summarizes cell wall components and their status at PD. Evidence of their contributions to cell wall mechanics and recent papers (last 3 years) reporting their relevance at PD are also presented

Cell wall component	Status at PD	Effects on mechanical properties*^a^*	Recent links to PD structure/function*^b^*
β-Glucans (callose and cellulose)	Enriched in callose, depleted in cellulose	Callose in pollen tubes controls stiffness, viscoelasticity, and cell turgor pressure (Parre and Geitmann, 2005; Kapoor and Geitmann, 2023). Cellulose increases cell wall stiffness and strength (Y. Zhang *et al.*, 2021; Cosgrove, 2022). Adding callose increases the elasticity and improves the ductility of cellulose gels (Abou-Saleh *et al.*, 2018; Kumari *et al.*, 2024).	Callose regulates PD during germline development (Somashekar *et al.*, 2024) (Pinto *et al.*, 2024), bud growth (Pan *et al.*, 2023), seed development (Wang *et al.*, 2023), vascular development (Z. Li *et al.*, 2024), and pathogen and abiotic stress responses (Tee *et al.*, 2023; Zhou *et al.*, 2023; Ayyoub *et al.*, 2024).
Xyloglucan (XG)	Depleted and highly fucosylated XG	Contributes with pectins to biomechanical hotspots regulating elongation (Park and Cosgrove, 2015). Fucosylation affects cellulose interactions, and cell wall strength and viscoelasticity (Vanzin *et al.*, 2002; Videcoq *et al.*, 2017)	Identified via PD cell wall enzymatic fingerprinting (Paterlini *et al.*, 2022). XG metabolic enzymes found at PD (Gombos *et al.*, 2023).
Pectin (Homogalacturonan, HG)	Low esterified HG	HG demethylesterification increases cell wall elasticity (Peaucelle *et al.*, 2011). De-esterified HG can associate with calcium and stiffen walls (Guo *et al.*, 2022)	Identified via PD cell wall enzymatic fingerprinting (Paterlini *et al.*, 2022). Pectin methylesterases affect virus spreading (Otulak-Koziel *et al.*, 2024). Mutation in pectin acetyl-transferase PMR5 increases cell wall stiffness and reduces number of secondary PD (Jay *et al.*, 2025)
Pectin (Rhamnogalacturonan, RG)	Low-substituted, RGI with arabinan side chain, highly methyl-acetylated RGII	RG structures are involved in cell adhesion (Yang *et al.*, 2020) and cell wall stiffness (Carroll *et al.*, 2022; Panter *et al.*, 2023).	Identified via PD cell wall enzymatic fingerprinting (Paterlini *et al.*, 2022). UDP-L-Rhamnose synthase (*SSM1/ROL1*) impacts cell wall RG content and PD aperture (Jacquier *et al.*, 2025)
Cell wall proteins	Arabinogalactan and expansin proteins found	Signalling cell wall modifications, loosening of cellulose fibres (Cosgrove, 2024)	Involved in PD biogenesis (Okawa *et al.*, 2023).

^*a*^Citations are provided as examples of work in this area. This is not a comprehensive list.

^*b*^Citations are relevant papers from the past 3 years provided as examples of work on this topic.

Transcription factors, signalling proteins, and RNAs move via plasmodesmata to regulate, non-cell autonomously, gene expression within tissues/organs (Bayer and Benitez-Alfonso, 2024; Tee and Faulkner, 2024; Zanini and Burch-Smith, 2024). In liaison with apoplastic transporters, such as sucrose and auxin carriers, symplasmic diffusion can also contribute to the formation of information gradients (i.e. uneven distribution of signalling molecules across neighbouring cells acting as messengers to regulate cell fate and development) (Band, 2021; Sankoh and Burch-Smith, 2021). Despite their importance, research on plasmodesmata regulatory mechanisms is lacking. Their small size and their location (embedded in complex cell walls containing large polymers) make difficult their visualization, analysis, and biochemical purification. Quantitative methods, to determine the plasmodesma aperture and permeability, are restricted to a few molecular probes and invasive techniques such as transmission electron microscopy (TEM). These limitations hinder the gathering of information on the factors that affect plasmodesmata regulation, including how they open, close, or selectively permit the passage of specific molecules.

Recent progress using experimental and computational methods, indicate the influence of geometrical (i.e. shape) and biophysical factors such as cytoplasmic streaming and osmotic pressure in the regulation of plasmodesmata connectivity and intercellular flow rate (Deinum *et al.*, 2019; Peters *et al.*, 2021; Ostermeyer *et al.*, 2022). Supported by proteomic and mutant screenings, the research also indicates that symplasmic permeability is dependent on plasmodesmata number (density per cell wall area), distribution (e.g. clustering), and on interactions between the plasma membrane and the desmotubule as well as on the width and composition of neighbouring cell walls (Kirk and Benitez-Alfonso, 2022; German *et al.*, 2023; Bayer and Benitez-Alfonso, 2024). Cell walls around plasmodesmata display accumulation of a minor hemicellulosic component named callose, a β-1,3-glucan (Amsbury *et al.*, 2018). Callose synthesis and degradation (turnover) occur directly at plasmodesmata sites. Altering callose homeostasis affects symplasmic permeability and the cell response to developmental/physiological signals and environmental cues such as wounding, drought, and pathogen attack. Both plasmodesmata development (branching, number, etc.) and regulation of its aperture/permeability, requires modifications in cell wall composition and properties but we lack detailed mechanistic understanding of these processes. It is also unclear how cell walls around plasmodesmata remain elastic to accommodate the transport of molecules, sometimes significantly larger than the observed pore aperture. For example, electron microscopy of Arabidopsis root meristems shows very narrow plasmodesmata pores [no more than a few nanometres or lacking a cytoplasmic sleeve (Nicolas *et al.*, 2017)]. Their size contrasts with their capacity to freely transport the green fluorescent protein (GFP), a 28 kDa protein with a length of 4.2 nm and a diameter of about 2.4 nm (Hink *et al.*, 2000; Peters *et al.*, 2021). A holistic picture is missing reflecting how cell wall microdomains (surrounding plasmodesmata) interact and integrate with the function of cell walls as major structural and functional barriers. It is also unknown how these microdomains remain flexible to regulate the formation, aperture, and branching of plasmodesmata during plant development.

The vast majority of research on cell walls surrounding plasmodesmata concerns the regulation of callose (Amsbury *et al.*, 2018). The influence of other cell wall components, cell wall integrity mechanisms and cell wall biomechanical properties remain obscure. This is in spite of recent evidence of callose interactions in cell walls (Abou-Saleh *et al.*, 2018; Bourdon *et al.*, 2023; Kumari *et al.*, 2024) and the identification of other enzymatic cell wall activities (such as xyloglucan endotransglucosylase/hydrolases, XET/XTH, and pectin methyl esterases, PME) in the plasmodesmata proteome (Gombos *et al.*, 2023). In this review, we present knowledge on cell wall components and their biomechanical properties. We bring together our current understanding of cell wall regulation with the aim to build a model that reflects its contribution to symplasmic intercellular transport. We discuss the challenge in analysing the mechanical properties of plasmodesmata *in situ* and highlight opportunities to use this knowledge in the development of resources to target plasmodesmata *in planta* and in the design of biomimetics inspired by their functional complexity.

## Plasmodesmata cell wall components and their mechanical properties

The occurrence of plasmodesmata dates a point within *Streptophyta* evolution, prior to the emergence of land plants (Brunkard and Zambryski, 2017). Plasmodesmata structural and functional analogues are present in all studied multicellular organisms with cellulosic cell walls. In *Chara*, plasmodesmata form during cytokinesis and adopt their characteristic structure with a central desmotubule and spokes between the desmotubule and the plasma membrane (Cook *et al.*, 1997). At the very same evolutionary timepoint, cell walls acquired an enhanced complexity, some charophytes displaying very similar components to those found in land plants today, containing hemicelluloses, calloses, pectins, and glycoproteins (Domozych and Bagdan, 2022). The evolutionary relationship between cell wall architecture and plasmodesmata complexity likely reflects a need to balance cell wall rigidity, mechanical function, and cell-to-cell connectivity. The formation of cell wall microdomains around plasmodesmata provides flexible control of plasmodesmata function without compromising whole cell wall biomechanics.

A combination of classical (e.g. immunolocalization, TEM, and proteomics) and more modern approaches (such as tomography) revealed insights on the composition of cell wall microdomains surrounding plasmodesmata (Amsbury *et al.*, 2018; Bayer and Benitez-Alfonso, 2024). More recently, enzymatic fingerprinting—a method where cell wall polysaccharides are digested with specific enzymes and the resulting fragments studied using Mass Spectrometry (MS)—identified components enriched in cell walls surrounding plasmodesmata isolated from cell cultures (Paterlini *et al.*, 2022). TEM reveals cellulose microfibrils in a circular arrangement and depleted in amount (and/or crystallinity) at plasmodesmata in relation to other cell wall regions (Fujita and Wasteneys, 2014). Plasmodesmata cell walls are enriched in pectin and callose, both of which have been shown to modify cellulose flexibility and degradability enabling plasmodesmata dynamic modifications in response to environmental and developmental cues (Johnson *et al.*, 2018; Alonso Baez and Bacete, 2023; Bourdon *et al.*, 2023; Cosgrove, 2024).

Some information concerning the mechanical properties of cell wall components is available but how these components specifically assemble and interact at plasmodesmata to determine its biomechanical properties is, in fact, unknown (Fig. 2, Table 2). *In vitro* and simulation studies have been used to predict interactions between callose and cellulose and their effects in solid or soft-polymer mechanics (Abou-Saleh *et al.*, 2018; Kumari *et al.*, 2024). In this section, we provide an overview on the main cell wall components of plasmodesmata (i.e. callose, pectin, xyloglucans, and cell wall proteins) highlighting recent reports on their structural and material properties. Dissecting the properties of one cell wall component without referring to another is challenging as polymer properties depend on their specific cell wall environment. Table 2 summarizes information including plasmodesmata cell wall composition, the mechanical properties ascribed to plasmodesmata components, and the evidence, where available, of their role in regulating symplasmic transport.

### The regulation and mechanical properties of callose

It is accepted that cell walls surrounding plasmodesmata are depleted in cellulose and enriched in callose (Table 2). Callose is found at low levels in primary cell walls but accumulates in pollen grains, at phloem sieve elements, at the cell plate, and in papillae that form in response to pathogen attack (Barratt *et al.*, 2011; Xie and Hong, 2011; Peters *et al.*, 2021; German *et al.*, 2023; Kapoor and Geitmann, 2023; Tee and Faulkner, 2024). Callose metabolic enzymes and proteins involved in its turnover localize at the plasmodesmata membranes and their effects on symplasmic transport are well documented (Kirk and Benitez-Alfonso, 2022; German *et al.*, 2023; Bayer and Benitez-Alfonso, 2024). Modifying callose has been shown to regulate the non-cell autonomous function of transcription factors, including FLOWERING LOCUS T (FT) and CENTRODIALS-LIKE1 (CENL1, which is the *Populus* orthologue of Arabidopsis TERMINAL FLOWER1) driving dormancy break and the transition to flowering in poplar (Rinne *et al.*, 2011). Callose accumulation also restricts the transport of the tomato transcription factor WUSCHEL interrupting a feedback loop responsible for fruit malformations after cold stress (Wu *et al.*, 2023). Other important biological processes, such as the formation of lateral root organs (Benitez-Alfonso *et al.*, 2013; Maule *et al.*, 2013; Gaudioso-Pedraza *et al.*, 2018), vascular development (Vaten *et al.*, 2011; Ohba *et al.*, 2023; Z. Li *et al.*, 2024), and fruit growth (Beauchet *et al.*, 2024), have also been linked to callose regulation at plasmodesmata. Research also highlights a role for callose in auxin, abscisic acid (ABA), gibberellins, and strigolactone signalling, in the response to drought (Ayyoub *et al.*, 2024) and in the spreading of viruses and other microbes (reviewed in German *et al.*, 2023; Bayer and Benitez-Alfonso, 2024; Tee and Faulkner, 2024 and references therein).

Family members of the GLYCOSYL HYDROLASES family 17 (GH17), also named BETA 1,3 GLUCANASES (BG) (enzymes with callose-degrading activity), and CALLOSE SYNTHASES (CALS), also known as GLUCAN SYNTHASE LIKE (GSL), were annotated in the plasmodesmata proteome of the bryophyte *Physcomitrium patens* (Gombos *et al.*, 2023), and in various angiosperms including *Arabidopsis thaliana* (Fernandez-Calvino *et al.*, 2011; Brault *et al.*, 2019), *Populus trichocarpa* (Leijon *et al.*, 2018) and *Nicotiana benthamiana* (Thomas *et al.*, 2008; S. H. Park *et al.*, 2017; Kirk *et al.*, 2022; Johnston *et al.*, 2023). Mutations in both CALSs and BGs have been reported in the past 2 years demonstrating their function in pollen development (Somashekar *et al.*, 2024), female gametogenesis (Pinto *et al.*, 2024), bud growth (Pan *et al.*, 2023), seed longevity (C. Wang *et al.*, 2023), and in response to the rice blight pathogen *Rhizoctonia solani* (Zhou *et al.*, 2023).

The plasmodesmata proteome is also rich in proteins that modulate callose deposition such as the PLASMODESMATA LOCATED PROTEINS (PDLP) (Thomas *et al.*, 2008; X. Chen *et al.*, 2024; Z. Li *et al.*, 2024) and the PLASMODESMATA CALLOSE BINDING (PDCB) protein family (Simpson *et al.*, 2009; W. Li *et al.*, 2023; X. Chen *et al.*, 2024). Recent research found NON-RACE SPECIFIC DISEASE RESISTANCE/HIN1 HAIRPIN-INDUCED-LIKE 3 (NHL3) and the CELL NUMBER REGULATOR FW2.2 proteins located at plasmodesmata where they interact with CALSs to regulate callose accumulation (Tee *et al.*, 2023; Ayyoub *et al.*, 2024; Beauchet *et al.*, 2024). Other proteins, such as an inositol phosphoryl ceramide synthase ERH1 influence callose deposition by altering membrane composition and the localization of BGs and PDCB proteins (Iswanto *et al.*, 2024). Examination and visualization by TEM of mutants defective in callose synthesis (e.g. the Arabidopsis *cals3m* mutant) show that accumulation of callose regulates plasmodesmata cytoplasmic aperture and length (Vaten *et al.*, 2011). Electron-dense regions enriched in gold particles after immuno-gold labelling callose were used as evidence of its accumulation around plasmodesmata (Nezhad *et al.*, 2013; Giannoutsou *et al.*, 2020).

The physical properties of callose were studied *in vivo* in pollen cell walls, where it regulates cell wall resistance to circumferential tension stress and cell turgor-equilibrium (Parre and Geitmann, 2005; Kapoor and Geitmann, 2023). During cytokinesis, callose is essential for the mechanical stability of the developing cell plate and for cell plate maturation guiding the deposition pattern of other cell wall polysaccharides (Drakakaki, 2015; Davis *et al.*, 2020). Analysis of biomimetic mixtures, using Nuclear Magnetic Resonance (NMR), Atomic Force Microscopy (AFM), and rheology revealed that callose interacts with cellulose acting as a plasticizer that increases gel ductility (see Table 1 for definitions) (Abou-Saleh *et al.*, 2018). *In silico* modelling of callose and cellulose mixtures support these results and revealed callose hygroscopicity as a driver for this physical behaviour (Kumari *et al.*, 2024). Interestingly, work in poplar secondary cell walls also points to callose as a modifier of cellulose macrofibrils (Bourdon *et al.*, 2023). In this context, increasing callose accumulation altered cell wall/cellulose crystallinity and water content improving enzymatic accessibility. Moreover, callose changed lignin polymerization around the macrofibril increasing lignin-cellulose distances and pore size. In contrast to our *in vitro* studies, callose accumulation in poplar wood had no significant effect on tensile strength or stiffness likely because of the compensatory effects on lignin polymerization (Bourdon *et al.*, 2023). Nevertheless, increased hygroscopicity and porosity was sufficient to improve saccharification of this cell wall material suggesting softening (Bourdon *et al.*, 2023). Although it is difficult to conclude if these properties are conserved in primary cell walls and/or at plasmodesmata, the results suggest interactions between callose and other cell wall components influencing plasmodesmata wall biomechanics and capacity for transport. These studies also highlight a hygroscopic behaviour for callose underpinning its role in controlling the elastic properties of cell walls and the plasmodesmata aperture. Callose accumulation around the channel might simply produce swelling (water uptake) in cell walls leading to passive blocking of plasmodesmata. This hypothesis could also explain the role of callose in the response to water/osmotic pressure and other chemical factors (such as pH or reactive oxygen species) induced in response to abiotic or biotic cues (Grison *et al.*, 2019; Hernández-Hernández *et al.*, 2020; Kirk *et al.*, 2022).

### Pectins and their co-regulation with callose at plasmodesmata

Besides callose, pectin structural modification appears to occur at plasmodesmata as inferred by the presence of PMEs, polygalacturonases, and other modifying enzymes in the plasmodesmata proteome (Kirk *et al.*, 2022; Johnston *et al.*, 2023; Bayer and Benitez-Alfonso, 2024). Antibody labelling (specifically arabinan-side chain epitopes detected by the LM6 antibody) indicates the presence of RGI pectins at plasmodesmata and at pitfields in the tomato fruit pericarp (Faulkner *et al.*, 2008; Amsbury *et al.*, 2018). Recent enzymatic fingerprinting of plasmodesmata-enriched cell wall fractions identified low-substituted RGI and acetylated RGII (Paterlini *et al.*, 2022). Recent determinations using stomata mutants indicate a role for highly fucosylated RGII and long linear arabinan-side chains RGI in the regulation of cell wall stiffness (measured using AFM) (Carroll *et al.*, 2022; Panter *et al.*, 2023). The functional significance of RG pectin structures at plasmodesmata is unknown but recent work revealed that significant reduction in RGI in the *Arabidopsis thaliana ssm1/rol1* pectin biosynthesis mutant correlates with an increase in symplasmic connectivity (Jacquier *et al.*, 2025). TEM analysis indicates an increase in plasmodesmata width in *ssm1*, likely due to changes in cell wall physico-mechanics, affecting plasmodesmata permeability and transport directionality.

The enzymatic fingerprinting experiment also identifies the presence of poorly methyl-esterified HGs (a product of PMEs, which are enzymes that remove methyl esters groups from HG) in plasmodesmata cell walls (Paterlini *et al.*, 2022). The localization of PMEs at plasmodesmata and their interaction with viral Movement Proteins (MPs) have been reported previously (e.g. M.H. Chen and Citovsky, 2003). According to Guo *et al.* (2022), HG pectins are secreted to plasmodesmata where they are demethylated and deacetylated. De-methylesterified HG can associate with calcium ions increasing cell wall density, adhesion, and mechanical strength (Guo *et al.*, 2022). Interactions between PMEs and MPs affect HG esterification and loosen cell walls and this activity seems important for the spreading of viruses via plasmodesmata (Otulak-Koziel *et al.*, 2024).

Pectins play a role in gelling cellulose microfibrils creating biomechanical hotspots (Fig. 1) of high mechanical strength that control cell wall extensibility, cell expansion, and structural integrity (Cosgrove, 2018). Modifications in pectin structures (including methyl esterification and acetylation) dynamically affect cellulose stiffness, and cell wall plasticity and porosity (M. Wang *et al.*, 2013). In a recent work, de-acetylation of pectins in cell walls was correlated with altered plasmodesmata number (M. Wang *et al.*, 2013). A mutation in *PMR5* (a pectin acetyl-transferase), led to a reduction in secondary plasmodesmata formation specifically in the epidermis–mesophyll interface, affecting the transport of small (s)RNA molecules. AFM nanoindentation was applied to dissected periclinal cell walls from Arabidopsis cotyledons. This method confirmed an increase in wall stiffness in the *pmr5* mutant linking pectin acetylation, cell wall extensibility, and the formation of secondary plasmodesmata.

Co-regulation of pectin and callose metabolism could determine plasmodesmata shape and function as similar mechanisms were described underpinning the differential distribution of callose and pectin epitopes in stomata cell walls (Giannoutsou *et al.*, 2020). Co-regulation between pectin degradation and the activation of callose synthesis was also described during sieve pore biogenesis as result of characterizing a *PECTATE LYASE LIKE* (*PLL*) gene in *Arabidopsis thaliana* (Kalmbach *et al.*, 2023). Interactions between callose and pectins to finely tune the properties of plasmodesmata cell walls is assumed but yet to be demonstrated.

### The role of xyloglucan and cell wall proteins in plasmodesmata regulation

Another cell wall component, recently identified by the enzymatic fingerprinting of isolated plasmodesmata cell walls, are fucosylated xyloglucans (Paterlini *et al.*, 2022). Xyloglucans, together with pectins, contribute to the formation of biomechanical hotspots through interacting with cellulose microfibrils (Fig. 1) (Y.B. Park and Cosgrove, 2015). The emergence of xyloglucans is associated with the terrestrialization of charophycean green algae (Del-Bem, 2018), coinciding with the evolution of novel cell wall architectures and of plasmodesmata in this lineage (Brunkard and Zambryski, 2017). Mutants in xyloglucan metabolism display defects in cell wall elasticity and rupture point (although these have been interpreted in light of compensatory effects on cellulose and pectin metabolism) (Sowinski *et al.*, 2022). An *A. thaliana* mutant of the *Cellulose synthase like-C* (*cslc*) gene was recently found to reduce xyloglucan production, elastic asymmetry, and turgor pressure which compromises its response to mechanical stress (Bou Daher *et al.*, 2024). Complementation analysis of the quintuple mutant indicates a role for these enzymes in xyloglucan synthesis (Kim *et al.*, 2020). Xyloglucan fucosylation appears to improve cellulose binding, thus potentially affecting biomechanical hotspots. Contradictorily, an Arabidopsis mutant lacking fucosylated xyloglucan did not show significant cell wall defects (Vanzin *et al.*, 2002). In apple, on the other hand, enzymatic digestion of xyloglucan fucosyl linkages had a detrimental effect on the storage modulus, which measures viscoelasticity (Videcoq *et al.*, 2017).

The role of xyloglucans at plasmodesmata is linked to the identification of XET/XTH enzymes in the proteomic analysis of moss *P. paten*s (Gombos *et al.*, 2023). These enzymes catalyse the hydrolysis and crosslinking of xyloglucans, but their expression can have indirect effects in other cell wall components. XET/XTH expression, regulated by biotic and abiotic stresses, impacts cell wall extensibility and cellulose crystallinity (Yan *et al.*, 2019; Fu *et al.*, 2024), thus these enzymes might be playing a role in secondary plasmodesmata formation.

Arabinogalactan proteins (AGPs) are also found at or near plasmodesmata cell walls where they appear to regulate plasmodesmata biogenesis (Okawa *et al.*, 2023). Immunogold experiments found the JIM13 antibody (which detects the AGP epitope) near plasmodesmata in pedestal cells of the carnivorous plant *Utricularia* (Plachno *et al.*, 2024). An Arabidopsis galactosyltransferases triple mutant (*hpgt1,2,3*) showed a reduction in AGPs and in cellulose but an increase in pectin and calcium leading to defects in cell wall porosity (Okawa *et al.*, 2023). The mutant displayed stomata patterning defects, which were linked to an increase in plasmodesmata conductivity and in the transport of the mobile developmental factor SPEECHLESS, previously shown to control stomatal development in *A. thaliana* (Guseman *et al.*, 2010). TEM analysis revealed the presence of a higher number of complex plasmodesmata structures with large cavities in the mutant versus the wild-type explaining the observed increase in conductivity. In addition to AGPs, the cell wall protein EXPANSIN 1 (EXPA1), promoted symplasmic transport via increasing plasmodesmata opening in tobacco (S.H. Park *et al.*, 2017). The effect of expansin in cell wall loosening is well characterized (Cosgrove, 2015, 2024), whereas other AGPs appear to have a stress signalling function in the regulation of wall thickness, cellulose content, and biomechanics (Ma and Johnson, 2023).

## The challenge of measuring plasmodesmata cell wall mechanics

The physical laws governing plasmodesmata transport has been deduced from the analysis of their geometrical parameters (such as radius, length, branching, clustering, and funnel aperture) (Peters *et al.*, 2021; Ostermeyer *et al.*, 2022) recently reviewed in Bayer and Benitez-Alfonso (2024). Studies also consider the effects of cytoplasmic streaming/flow and turgor pressure on desmotubule positioning (K. Park *et al.*, 2019), plasmodesmata conductivity, and transport directionality (K. Park *et al.*, 2019; Hernández-Hernández *et al.*, 2020). Hernández-Hernández *et al.* (2024) report on how pressure differences and cytoplasmic flow drives symplasmic transport through diffusion and advection mechanisms and discuss feedback relationships between callose and turgor pressure connecting cell wall dynamics and plasmodesmata mechanohydraulics. Further, computational models emerged improving understanding of plasmodesmata mechanohydraulics and its importance to cell wall elongation (Hernández-Hernández *et al.*, 2024). They were also used to establish relationships between ultrastructural data on plasmodesmata geometry and permeability factors extracted experimentally using transport assays (Deinum *et al.*, 2019; Peters *et al.*, 2021). The studies above predict a clear link between turgor pressure, cell wall regulation, and symplasmic transport but, as far as we know, the mechanical properties of plasmodesmata cell walls have not been experimentally measured. This is not a trivial task due to the small size of these domains challenging the resolution of most mechanical testing protocols. Without understanding the biomechanical properties of plasmodesmata walls, and how they change in response to the whole cell/tissue dynamics during development, it is difficult to piece together all the information required to predict and engineer plasmodesmata conductivity.

New developments have provided tools to investigate the properties of plasmodesmata *in situ* and/or *in vivo* (Alonso Baez and Bacete, 2023). Advances in AFM-nanoindentation improved the structural resolution at the nanoscale level enabling their use, in isolation or in combination with other imaging protocols such as fluorescence-lifetime imaging microscopy (FLIM), in the study of the structural and mechanical properties of cell wall microdomains (Pu *et al.*, 2025). In addition, Brillouin microscopy (a technique based on the scattering of light after interaction with the sample’s natural thermal vibrations, known as acoustic phonons) together with Raman imaging emerges as a powerful non-invasive method to simultaneously scan tissue mechanics (Brillouin shift, defined as a microscopic high-frequency longitudinal modulus) and composition (J. Zhang and Scarcelli, 2021; Alonso Baez and Bacete, 2023). Another imaging platform, the automated confocal micro-extensometer (ACME), exploits the resolution of confocal microscopy to allow mechanical determinations (elasticity, creep, and yield stress) in cells within tissues (Robinson *et al.*, 2017). Most of these methods are limited by the lack of mathematical frameworks that distinguish microdomains within the same cell wall (surrounding and far from plasmodesmata). This issue was recently overcome by the development of new molecular rotors (CarboTags) to target plasma membrane and cell wall domains, including plasmodesmata, enabling visualization of their properties *in vivo* (Besten *et al.*, 2025).

Unfortunately, not all cell wall properties can be measured with a single tool. While AFM nanoindentation informs on cell wall stiffness (i.e. Young’s modulus, defined as the resistance to elastic deformation or the relationship between force versus displacement), ACME applies a tensile test measuring extension and shear deformation of cells/tissues under applied force. CarboTags, on the other hand, report on membrane fluidity and cell wall porosity. Therefore, results from analysing cell walls are not comparable, and they might appear contradictory. Physical and mathematical modelling approaches are expected to play a key role in addressing this challenge by placing all cell wall components (and their structural variations) into context allowing for predictions on mechanical parameters that can be compared with the experimental values (Pieczywek *et al.*, 2023). Recently, Y. Zhang *et al.* (2021) developed a coarse-grained model to recapitulate some of the structural and mechanical parameters observed in onion epidermal cell walls. Similarly, Smithers *et al.* (2024) proved, using continuum mechanical modelling, that biomechanical hotspots are formed in regions where cellulose interacts with xyloglucans and explained how they might control growth after enzymatic action. Advances of this nature will improve our understanding of the importance of cell wall composition in intercellular (and particularly symplasmic) transport where a link between architecture, mechanical properties, and function is still missing.

## Conclusion

Cumulative evidence suggests that cell walls surrounding plasmodesmata are special in that they contain callose, reduced cellulose, structural substitutions in pectins and xyloglucans, and a subset of cell wall proteins. It is yet unknown if structural differences are conserved in all plasmodesmata, how they emerged throughout plant evolution, and how they are modified in response to developmental and environmental factors. Interactions between these cell wall components are expected but the effect this has on cell biomechanics and symplasmic intercellular transport remains to be determined.

Questions remain regarding the factors that determine the biomechanical properties of plasmodesmata and their impact on cell responses to environmental stresses (see Box 1). Future years will see results of applying a combination of structural biology and mechanical testing methods (currently under development) to answer some of these questions leading to a better more holistic understanding of plasmodesmata function and regulation. The gaining of knowledge on the mechanical behaviour of cell wall components in the plasmodesmata micro-environment may lead to new predictions to target these structures and their functioning in plant growth, during plant adaptation to new environments, and as a defence mechanism. It may also uncover new polymer structures for bio-inspired materials. Success in achieving this goal will open exciting opportunities for biotechnological approaches to improve crop productivity and climate resilience, and for the design of sustainable cell wall bioproducts.

Box 1. **Outstanding questions in plasmodesmata cell wall biomechanics**

- How do the composition and mechanical properties of cell walls impact plasmodesmata ultrastructure, architectures/geometries?
- What are the main differences in the structural properties of plasmodesmata cell walls when comparing plant species, tissues, and/or organs, and how these are modulated in different environmental conditions?
- How do physiological parameters, such as water content and sugar transport/accumulation, influence, and are influenced by, the mechanical properties of plasmodesmata cell walls?
- Which biomechanical parameter best describes plasmodesmata wall behaviours and how they interact with other biophysical parameters, such as desmotubule positioning or membrane fluidity, to impact symplasmic conductivity?
- How are developmental and/or environmental alterations in plasmodesmata walls mechanistically translated to affect plant cell/tissue/organ response to stress?
- Do changes in the composition and mechanics of plasmodesmata cell walls impact apoplastic transport, molecular cell wall diffusion, and cell adhesion?
